# Metabolic Rewiring in Cancer: Small Molecule Inhibitors in Colorectal Cancer Therapy

**DOI:** 10.3390/molecules29092110

**Published:** 2024-05-02

**Authors:** Domiziana Masci, Michela Puxeddu, Romano Silvestri, Giuseppe La Regina

**Affiliations:** 1 Department of Basic Biotechnological Sciences, Intensivological and Perioperative Clinics, Catholic University of the Sacred Heart, Largo Francesco Vito 1, 00168 Rome, Italy; domiziana.masci@unicatt.it; 2 Laboratory Affiliated to Istituto Pasteur Italia-Fondazione Cenci Bolognetti, Department of Drug Chemistry and Technologies, Sapienza University of Rome, Piazzale Aldo Moro 5, 00185 Rome, Italy; michela.puxeddu@uniroma1.it (M.P.); romano.silvestri@uniroma1.it (R.S.)

**Keywords:** metabolism, Warburg effect, lactate dehydrogenase, lipid metabolism, glutaminolysis, cancer, colorectal cancer

## Abstract

Alterations in cellular metabolism, such as dysregulation in glycolysis, lipid metabolism, and glutaminolysis in response to hypoxic and low-nutrient conditions within the tumor microenvironment, are well-recognized hallmarks of cancer. Therefore, understanding the interplay between aerobic glycolysis, lipid metabolism, and glutaminolysis is crucial for developing effective metabolism-based therapies for cancer, particularly in the context of colorectal cancer (CRC). In this regard, the present review explores the complex field of metabolic reprogramming in tumorigenesis and progression, providing insights into the current landscape of small molecule inhibitors targeting tumorigenic metabolic pathways and their implications for CRC treatment.

## 1. Introduction

Colorectal cancer (CRC) is the second leading cause of cancer-related death in women and the third most common cause in men, with more than 1.9 million new CRC cases worldwide being estimated in 2020 [1]. CRC risk factors include a combination of genetic, lifestyle, and environmental factors. The age of a patient represents a major risk factor for sporadic CRC, with an increasing incidence rate after 50 years of age [2]. Adenomatous polyposis and Lynch syndrome (also known as hereditary nonpolyposis colorectal cancer) account for the most common familial CRC cases (about 5% of CRC cases) [3,4,5]. Alcohol consumption [6] and cigarette smoking (3) have been associated with an increased incidence of CRC, whereas daily food consumption has been associated with a small absolute risk [2]. Cholecystectomy [7], orchidectomy [8], and gonadotropin-releasing hormone (GnRH) agonists [9] are also important conditions contributing to CRC development.

Treatment for CRC is primarily contingent on the cancer’s stage, reflecting the extent of its progression. The earliest stage of non-metastatic CRC, called stage 0 (i.e., tumor limited to the bowel or local lymph nodes, with no metastatic dissemination to distant organs), and stages I to III are mainly treated with radiotherapy and/or chemotherapy, whereas patients with stage IV CRC (metastatic CRC (mCRC)) are regularly treated with systemic chemotherapy, as it aims to treat cancer cells throughout the body. In addition, patients with specific genetic mutations, such as Kirsten rat sarcoma viral oncogene homolog (KRAS) or B-Raf proto-oncogene (BRAF) mutations, can be treated by combining targeted therapy with chemotherapy. Radiation therapy can be employed to effectively manage the disease and alleviate symptoms, such as pain or bleeding.

Based on 2017–2019 data, approximately 4.1% of the population is estimated to develop CRC at some point during their lifetime. The relative survival rate of 5 years or more after being diagnosed with CRC is 65%, whereas if CRC has spread to distant parts of the body, the 5-year relative survival rate drastically decreases to 13% [10].

While the past decade has witnessed a decline in mortality from colorectal cancer, thanks to both the advancements in screening techniques enabling earlier diagnosis and the adoption of clinical practice guidelines to streamline cancer care, a significant number of patients initially present with metastatic disease. In fact, about 25% of patients present with de novo mCRC, and up to 50% of individuals, even after receiving adjuvant treatment following surgery, develop mCRC [11].

Clinical studies have established a correlation between altered whole-body metabolism and cancer development stemming from common physiological alterations that not only occur within cancer cells, but also influence the biological behavior of nearby non-tumor cells [12]. Notably, altered cell metabolism, leading to an increased energy demand to support and accelerate abnormal cell proliferation, stands as one of the main hallmarks of tumorigenesis [13,14]. In this context, the prompted aerobic glycolysis process, also called the Warburg effect, has been recognized as an established metabolic feature of cancer cells [14,15]. However, the latter alone may not comprehensively depict the altered cellular metabolism in CRC; therefore, it is essential to consider cancer-specific metabolism arising from impaired mitochondrial function and an abnormal expression of drug-metabolizing enzymes (DMEs).

In this review, we depict some of the dysregulated metabolic pathways, which have made a significant contribution in the development and progression of cancer, with a specific focus on small-molecule metabolic inhibitors that have shown potential for application in the management of CRC.

## 2. Metabolism-Based Inhibitors

### 2.1. Glycolytic Metabolism in Cancer

**Figure 1:**
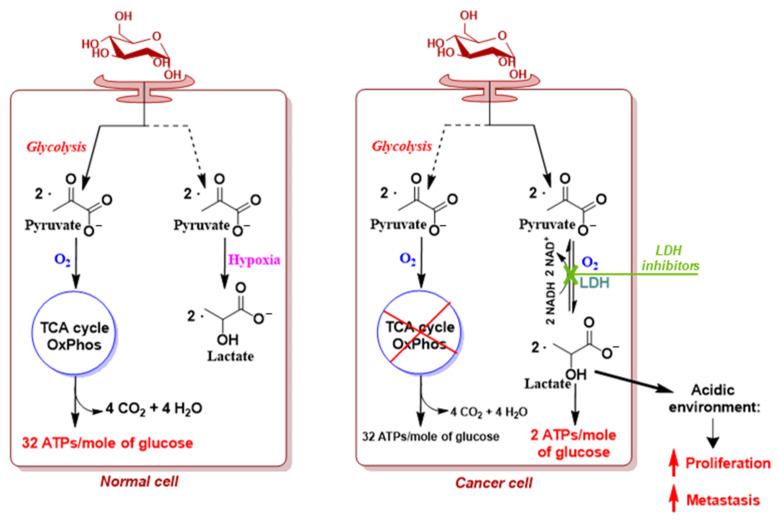
Schematic representation of “Warburg effect” in cancer cells.

The link between tumorigenesis and altered cancer cell metabolism was first described a century ago by the German biochemist Otto Warburg [16], who observed, while working with a rat seminal vesicle tumor, that cancer cells actively use glycolysis for adenosine triphosphate (ATP) generation, even in the presence of an abundant supply of oxygen, bypassing oxidative phosphorylation (OxPhos). Indeed, he demonstrated that, in malignant tumors, most of the glucose was rapidly converted into lactate through the activation of lactate dehydrogenase, resulting in uncontrolled cancer cell proliferation [17,18] (Figure 1).

This phenomenon, which was later observed even in the presence of completely functioning mitochondria, thereby questioning Warburg’s hypothesis of mitochondrial and OxPhos chain damage being a reason for cancer transformation, was termed the Warburg effect or aerobic glycolysis by Efraim Racker and represents a distinguishing feature observed in almost all types of cancers, including CRC.

After years of debate and investigation, the Warburg effect has recently been incorporated into the broader spectrum of tumor cell metabolic reprogramming irrespective of oxygen levels. This led to a loss of tumor suppressors’ function, tumor microenvironment remodeling, the alteration of signaling pathways, the overexpression of hypoxia-inducible factor 1 (HIF-1)—a transcription factor responsible for oxygen homeostasis—and interference with epigenetic mechanisms [19].

Considering the crucial role played by the Warburg effect in indicating malignant tumor transformation, our aim in the present review is to provide a concise analysis of the strategies that modulate this phenomenon, highlighting its potential significance in the development of therapeutic approaches for cancer treatment by targeting some key enzymes involved in this process.

#### Lactate Dehydrogenase Inhibitors

**Figure 2:**
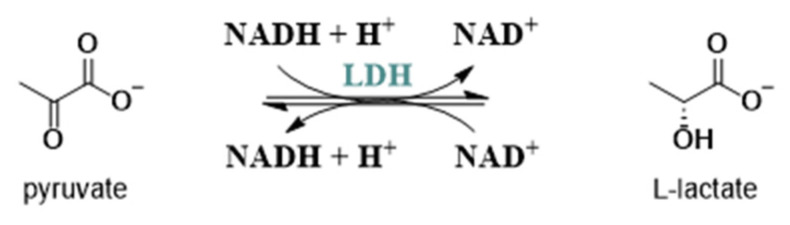
Reaction catalyzed by LDH.

Lactate dehydrogenase (LDH, EC 1.1.1.27) is an important cytoplasmatic enzyme that is able to catalyze the reversible conversion of pyruvate into lactate with the simultaneous oxidation/reduction of nicotinamide adenine dinucleotide (NADH) to NAD^+^ (Figure 2). It exists in five human isoforms (hLDH1 to hLDH5) assembled in tetramers and is composed of two different types of subunits: subunit M, encoded by lactate dehydrogenase-A (*LDHA*) gene, which is predominately found in skeletal muscle and the liver, and subunit H, encoded by lactate dehydrogenase-B (*LDHB*) gene, which is found in the heart muscles, brain, spleen, kidney, and erythrocytes. The 4M tetrameric form LDHA, due to the higher affinity to pyruvate, catalyzes the conversion of the latter to lactate during cell aerobic glycolysis; therefore, its aberrant expression and activation have been found to be related to different types of cancers [20,21], whereas LDHB controls the oxidation of lactate to pyruvate in the process of cell aerobic glycolysis in the heart and brain.

The distinctive function of lactate dehydrogenase becomes notably evident under oxygen-limited conditions, where the oxidation of NADH in the respiratory chain is prevented. Therefore, LDH catalyzes the reduction in pyruvate, facilitating the replenishment of NAD^+^ molecules, which are essential for the ongoing production of ATP to support glycolysis. In this context, it should be also noted that the conversion of pyruvate to lactate, coupled with the accumulation of lactate produced by high glycolytic activity, induces an acidic state within the tumor immune microenvironment. Concurrently, the increased energy requirements for tumor cell proliferation contribute to a microenvironment characterized by a low oxygen level and a reduced energy level. Therefore, the resulting hypoxic, low-pH, and low-energy microenvironment significantly influences the human immune system, producing immunosuppressive factors, affecting T cell functionality, and promoting tumor invasion and metastasis [22,23,24].

Notably, since it has been demonstrated that LDH is released from cells in response to cell damage, serum LDH is used in oncology as a prognostic biomarker for cancer patients. Indeed, it represents a multifaceted biomarker linked to the activation of several oncogenic signaling pathways and it exerts an influence on metabolic activity, invasiveness, and immunogenicity across a spectrum of tumors. Several studies have shown a change in the activity of LDH isoenzymes near the tumor area, even in the absence of visible morphological alterations [25]. In serum samples collected from adults without cancer, LDH2 is the prevailing isoenzyme, whereas LDH4 and LDH5 are less abundant [26]. Accordingly, it has been observed that an increase in serum LDH levels above 140–245 IU/L, along with the emergence of a rising hypoxic microenvironment, is associated with an unfavorable prognosis among patients with CRC who are undergoing chemotherapy with an antiangiogenic agent [24].

Therefore, considering the pivotal role played by LDHA in cancer growth, angiogenesis, metastasis, and drug resistance (e.g., tamoxifen resistance), it was assumed that LDH could be an extremely attractive target for the development of small molecules as anticancer agents [27]. Furthermore, recent reports suggest that the use of LDH inhibitors is likely to be safe due to the lack of clinically serious side effects related to LDH deficiencies in patients [24].

In this section, we present different LDHA inhibitors discovered so far, some of which have entered preclinical trials, while a few have entered clinical trials. Unfortunately, none have yet received approval.

**Figure 3:**
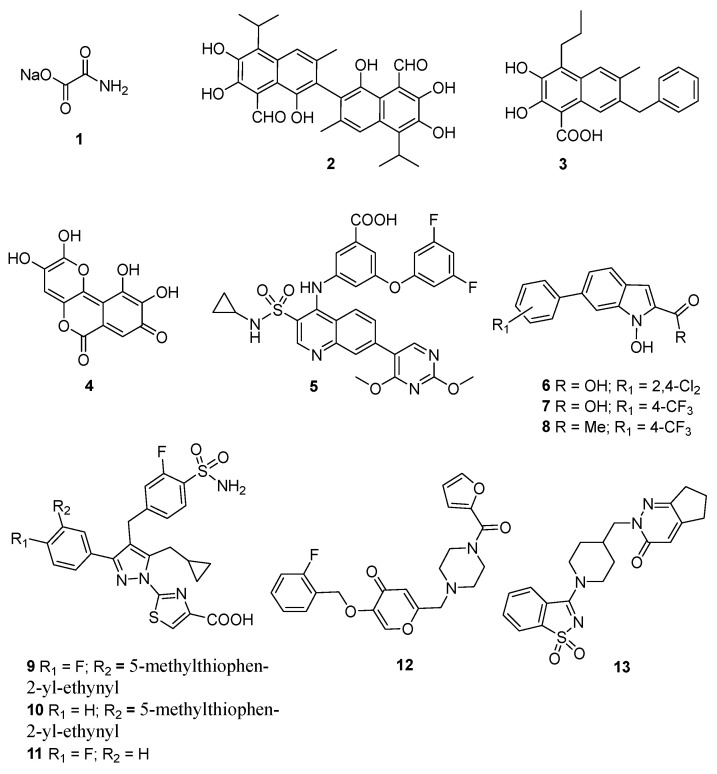
Chemical structures of lactate dehydrogenase inhibitors **1**–**13**.

1. Sodium oxamate (**1**) (2-amino-2-oxoacetic acid sodium salt, Figure 3) is a structural analogue of pyruvate that is able to inhibit both LDHA/LDHB and aspartate aminotransferase (AAT). It competitively inhibits human LDHA (K_I_ = 136 μM), binding at the pyruvate site and leading to the formation of an inactive complex [28]. Notably, several studies have shown the anticancer properties of compound **1** on different cancer cell lines, including those obtained from CRC, breast cancer, ovarian cancer, and lymphoma, highlighting the correlation with the inhibition of aerobic glycolysis due to its ability to significantly inhibit the growth of cancer cells exclusively under glucose-treated conditions [29,30,31,32,33]. In addition, in gastric cancer, where an overexpression of LDHA has been confirmed, sodium oxamate was observed to decrease lactate production, resulting in a dose-dependent inhibition of cancer cell proliferation in the presence of glucose.

In 2014, Yang et al. investigated the different effects of compound **1** on LDHA inhibition in non-small-cell lung cancer (NSCLC) cells. While previous studies reported that the inhibition of LDHA, exerted by compound **1** in H1395 cells, induced cells arrested in the G_2_/M cycle and subsequent apoptosis, Yang et al. first revealed that oxamate was able to induce autophagy in human non-small-cell lung cancer A549 cells. Moreover, in the presence of 3-MA, an autophagy inhibitor, compound **1** induced apoptosis, indicating that autophagy served as a protective mechanism against apoptosis and contributed to the drug resistance of LDHA inhibitors. Building on the knowledge that autophagy is involved in both glucose metabolism and the maintenance of malignancy in lung tumors, their findings suggest that the concurrent inhibition of autophagy represents a promising strategy to overcome drug resistance in NSCLC cells treated with LDHA inhibitors [34,35].

Interestingly, Salgado-García et al. recently investigated the pharmacological combination of compound **1**, metformin, and doxorubicin (DOX) on CRC cells, revealing the induction of the autophagy process and, subsequently, apoptosis [36]. This effect was attributed to the modulation of ULK1 protein levels, which are involved in the early initiation of autophagy, via the oncogenic microRNA-106a (miRNA-106a) [37,38]. In fact, when HCT116 CRC cells were treated with the three abovementioned drugs, the expression of miR-106a dropped and ULK1 was expressed, and thus, the autophagy process was initiated. In addition, due to the importance of autophagy in cancer biology, several studies have revealed, through real-time RT-PCR and microarray assays, the overexpression of miR-106a in plasma, cancer tissues, and stool samples of patients affected by CRC. Notably, since miRNA-106a triggers the progression of CRC, its presence has been suggested as a diagnostic marker for CRC [39].

Although **1** represents a useful tool for pharmacological studies, its weak potency, selectivity, and poor cellular uptake hamper its clinical utilization; it is therefore not a good candidate for clinical development [40]. Therefore, additional LDH inhibitors have been developed to overcome the limitations of compound **1**.

2. Gossypol (**2**) ((2,2′-binaphthalene)-8,8′-dicarboxaldehyde,1,1′,6,6′,7,7′-hexahydroxy-5,5′-diisopropyl-3,3′-dimethyl, Figure 3) is the most important natural polyphenolic pigment present in cotton (*Gossypium hirsutum* L.); first isolated in its pure form by Marchlewski in 1899 [41], it is the only compound with anti-LDH activity that has been evaluated clinically to date. Originally evaluated for use as a male antifertility agent, **2** showed broad-spectrum antitumor, antiviral, antibacterial, and antioxidant activities [42]. Despite displaying a wide array of potential biological activities, compound **2** has a poor applicability in drug development due to its toxicity, which is possibly attributed to the presence of reactive aldehyde and catechol hydroxyl functional groups [43].

It represents a non-selective LDH inhibitor with K_i_ values of 1.9 and 1.4 µM in LDHA and LDHB, respectively. In addition, it is a non-competitive inhibitor against substrates in LDH-induced oxidation, with an IC_50_ value of 9.8 μM when using pyruvate and 11.3 μM when using α-ketobutyrate as a substrate, whereas it is a strong competitive inhibitor against cofactors such as NADH [44]. Indeed, compound **2** was found to be a potent inhibitor of the NAD-linked enzymes LDH, glucelaldehyde-3-phosphate dehydrogenase (GA3PDH), malate dehydrogenase (MDH), and isocitrate dehydrogenase (ICDH). MDH was inhibited by gossypol in a concentration-dependent manner with an IC_50_ value of 2.9 μM in the direct (malate–oxalacetate) reaction and 1.2 μM in the reverse (oxalacetate–malate) reaction. In the GA3PDH and ICDH, **2** yielded IC_50_ values of 110 μM and 2.7 μM, respectively [42].

Gossypol exhibited a half-maximal inhibitory concentration of 50 μM in cell viability assays conducted on various cancer cell lines, including melanoma, leukemia, and cervical and breast cancers. Upon oral administration at a dose of 30–70 mg per day, compound **2** was generally well tolerated and showed potential to be used for the treatment of metastatic adrenal cancer [45], malignant glioma [46], and anthracycline- and taxane-resistant metastatic breast cancer [47]. However, several studies highlighted that single therapy with compound **2** might not be effective enough to induce cancer cell deaths due to the limited therapeutic response.

In addition, since gossypol acts as a non-specific LDHA inhibitor, its anticancer effects may be attributed to alternative mechanisms. This is evident in its modulation of various proteins associated with the cell cycle and apoptosis, including BCL-2; caspases 3, 6, 7, 8, and 9; as well as cyclin D1 [48].

3. FX11 (**3**) (3-dihydroxy-6-methyl-7-(phenylmethyl)-4-propylnaphthalene-1-carboxylic acid, Figure 3) is a synthetic drug-like small molecule that is structurally related to gossypol, which was initially designed as an antimalarial agent and discovered through a Heparin-induced thrombocytopenia (HIT) assay. It is well known that compound **3** represents a sensitizer in chemotherapy-resistant tumor cells, and it acts as an NADH-competitive, -selective, and -reversible hLDH5 inhibitor with a K_i_ value of 0.05 μM [20]. Furthermore, preclinical studies have shown its efficacy in inhibiting the progression of various adult cancers, including pancreatic cancer, prostate cancer, lymphoma, and osteosarcoma.

In 2010, Le et al. reported that the inhibitory activity of compound **3** is associated with a reduction in ATP levels, resulting in the induction of bioenergetic and oxidative stress and cell death, which could be partially reversed by *N*-acetylcysteine, the *N*-acetylated derivative of the amino acid L-cysteine [49]. In addition, they thoroughly described its ability to inhibit the progression of human lymphoma and pancreatic tumors in xenograft models. Notably, compound **3** induced the regression of lymphoma in combination with *Daporinad* (FK866 or APO866), a metabolic inhibitor of nicotinamide phosphoribosyltransferase (NAMPT), which is an enzyme involved in the biosynthesis of nicotinamide adenine dinucleotide (NAD^+^) from nicotinamide. In addition, compound **3** showed potential to be used for the treatment of LDHA-dependent tumors [49], inhibiting cell proliferation, migration, and invasion and promoting the cell apoptosis of DU145 and PC3 cells [50,51]. In this respect, Rajeshkumar et al. were the first to establish a connection between the p53 status of tumors and the sensitivity to LDH-A inhibitors. Specifically, they recently demonstrated that only in pancreatic cancer PDXs with mutant TP53, which is characterized by a higher glucose uptake compared to wild-type TP53 tumors, FX11 was able to inhibit the pyruvate-to-lactate conversion. The heightened sensitivity of mutant TP53 tumors to compound **3** was correlated with a reduced expression of the TP53-Induced Glycolysis Regulatory Phosphatase (TIGAR) target gene, which regulates glycolysis [52].

4. Galloflavin (**4**, Figure 3), a flavone-like compound synthesized by gallic acid, is a non-selective inhibitor of both LDHA and LDHB that preferentially binds to the free enzyme without competing with the substrate (pyruvate) or with the cofactor (NADH). The calculated K_i_ values for its inhibitory activity were 5.46 μM and 56.0 μM against LDHA and LDHB, respectively. Notably, its ability to inhibit LDHA was found to be beneficial in overcoming acquired resistance to taxol and trastuzumab in breast cancer cells, which are both drugs that are widely used to treat this disease. Moreover, **4** showed the inhibition of aerobic glycolysis, without interference with cell respiration, with the consequent reduction in the growth of different cell lines such as colon, breast, liver, Burkitt lymphoma, and endometrial cancer cells [53]. Compound **4** exerted a similar growth inhibition in MCF-7, MDA-MB-231, and MCF-Tam cells, for which high glucose uptake has been observed. MDA-MB-231 and MCF-Tam cells, which exhibited different LDH expression levels, also experienced growth inhibition due to a constitutively activated stress response. Farabegoli et al. emphasized that the antiproliferative effect of compound **4** against different cell lines was attributable to different mechanisms, such as the downregulation of the ERα-mediated signaling pathway in MCF-7 cells and the induction of an oxidative stress condition in MDA-MB-231 and MCF-Tam cells [54].

Interestingly, in a recent study conducted by Guo et al. [55], it was reported that in the inflammatory microenvironment, NLRP3 inflammasomes play a crucial role in promoting the metastasis and invasion of CRC cells, significantly contributing to the progression of CRC. Indeed, they proved the ability of compound **4** to exert an inhibitory effect on CRC progression by targeting NLRP3. These findings suggest that compound **4** might be a promising novel drug for CRC treatment, warranting further development.

5. 3-((3-carbamoyl-7-(3,5-dimethylisoxazol-4-yl)-6-methoxyquinolin-4-yl) amino) benzoic acid (GSK 2837808A, **5**, Figure 3) is a quinoline 3-sulfonamides-based compound, obtained following lead optimization studies, that exhibits an inhibitory potency against LDHA of less than 2 nM and has selectivity over LDHB ranging from 10 to 80-fold. Its effect is exerted in the rate of lactate production in different cancer cell lines, including hepatocellular and breast carcinomas [26]. In hepatocellular carcinoma cells (HCCs), Billiard et al. demonstrated the ability of compound **5** to increase oxygen levels at doses of up to 3 μM in HCCs, while at a higher concentration, mitochondrial function was directly inhibited. In addition, in wider terms, a metabolite analysis in Snu398 cells, upon treatment with a quinoline-3-sulfonamide LDHA inhibitor, revealed an increased intracellular concentration of intermediates of glycolysis and the citric acid cycle, which was consistent with the enhanced activity of the Krebs cycle and with the blockage of cytosolic glycolysis. This resulted in an enhanced activity of pyruvate kinase isozymes M2 (PKM2) and in the induction of apoptosis in Snu398 cells [50,51].

Moreover, in their work, Billiard et al. identified compound **5** as an NADH-competitive LDHA inhibitor. In fact, in a NADH-competitive model, compound **5**, showed to hinder cofactor binding [50]. The crystal structures of the human isoform 5 of LDH (hLDH5), which is detected in highly invasive and hypoxic carcinomas, confirmed that, in general, quinoline 3-sulfonamides-based compounds are not competitive compared to pyruvate, and their binding site is in the NADH pocket.

6. *N*-Hydroxyindole-2-carboxylate derivatives (NHIs, **6**, Figure 3). There has recently been a growing interest in developing NHIs as potential therapeutic agents due to their low likelihood of generating reactive intermediates [56,57]. Granchi et al. [58] reported the design and synthesis of a selected series of NHIs, with a particular focus on their inhibitory activity against LDHA, particularly targeting hLDH5. The importance of targeting the isoform 5 of LDHA is gathered from studies on LDHA-deprived cancer cell lines suggesting that targeting hLDH5 can significantly prevent tumor growth and invasiveness, especially under hypoxic conditions [59,60]. In addition, studies on individuals with hereditary LDHA deficiency revealed that the lack of this enzyme did not cause symptoms under normal circumstances, hence supporting the notion that hLDH5 could be a safe target for anti-tumor agents.

Since NHIs proved to sensibly decrease cellular lactate production, which is involved in the growth and survival of hypoxic tumors, Granchi et al. decided to further expand SAR studies by introducing chemical variability at the aryl substituents of the NHIs’ central nuclei [58]. Among the developed substituted derivatives, compounds **6** and **7**, bearing a 2,4-dichlorophenyl and a 4-(trifluoromethyl)phenyl moiety, respectively, at position 6 of the NHIs’ indole ring, exhibited potent and selective inhibition against hLDH5, although minimal activity against hLDH1 was kept [56]. Moreover, enzyme kinetics studies confirmed the competitive behavior of these compounds vs. both a pyruvate substrate and an NADH cofactor [61]. A few years later, Daniele et al. investigated the LDHA inhibitory effect of compound **6** and its methyl ester **8** (Figure 3) in different glioblastoma (GBM) cell lines and in cancer stem cells (CSCs). In their study, Daniele and coworkers [62] demonstrated the LDHA inhibitory effect performed by compounds **7** and **8**, in CSCs isolated from different GBM cell lines, through the activation of both apoptosis and cellular differentiation, resulting in the decrease in GBM proliferation. These data highlight the particular sensitivity of GBM-derived CSCs to glycolytic inhibition.

7. Pyrazole-based derivatives. In 2020, structure-guided SAR optimization studies of pyrazole-based derivatives [63] led Rai and coworkers [64] to the identification of two lead compounds, named NCATS-SM1440 (**9**, Figure 3) and NCATS-SM1441 (**10**, Figure 3), with robust in vivo LDH inhibition. Indeed, both compounds showed nearly identical potency against both LDHA and LDHB, with an IC_50_ value of 0.04 μM. Notably, their impact on the glycolytic pathway was assessed in human Ewing’s sarcoma (A673) cell lines through the glycolysis stress test (GST), which measures the extracellular acidification rate (ECAR) of the media. In this assay, the decrease in ECAR, which results from glycolysis-dependent proton production by the cells, indicated a depletion of NAD^+^ and, consequently, an inhibition of the entire pathway.

After achieving sub-micromolar cellular potency and favorable pharmacokinetic (PK) properties, Rai et al. evaluated compound **10** in a mouse tumor model using A673 cells for in vivo LDH inhibition [64]. Intravenous administration at 50 mg/kg of compound **10** led to a sustained reduction in LDH activity in tumors, as analyzed at 1 and 6 h post dose. Consistent with these findings, compound **10** also demonstrated good potency in both sarcoma and pancreatic xenograft models at similar doses.

Furthermore, it is worth mentioning that Chinook Therapeutics (a Novartis Company, Basel, Switzerland) recently developed a novel analogue of compound **10** called CHK-336 (**11**, Figure 3), which showed potent and selective LDHA inhibition with the potential for once-daily oral dosing [65]. In the liver, compound **11** targets the primary site of oxalate production and minimizes extra-hepatic LDH inhibition. In preclinical studies, **11** significantly reduced the urinary oxalate in a primary hyperoxaluria type 1 (PH1) mouse model compared to wild-type mice.

Therefore, compound **11** has been suggested as a first-in-class LDHA inhibitor and is being developed for the treatment of primary and idiopathic hyperoxaluria [65].

8. Pyran-4-one derivatives. The screening of a proprietary compound library containing 5000 small molecules led to the identification of 16 pyran-4-one-based compounds endowed with anti-LDHA activity. Among them, compound **12** (Figure 3), identified through hit-to-lead optimization studies by Zhou and co-workers [66], emerged as a potent LDHA inhibitor with an EC_50_ value of 90 nM.

Furthermore, it was shown to reduce the cancer cell growth of MiaPaCa-2 and A549 cells, with IC_50_ values of 2.1 and 7.8 μM, respectively [66]. In a MiaPaCa-2 cells xenograft model, **12** suppressed tumor growth at a dose of 10 mg/kg with little effect on the mice’s weights. In MiaPaCa-2 cells, **12** induced apoptosis, caused cell cycle arrest in the G_2_ phase, and inhibited MiaPaCa-2 cell colony formation in a dose–response manner. Furthermore, a mitochondrial bioenergetics analysis indicated that compound **12** redirected cancer cell metabolic pathways from glycolysis to oxidative phosphorylation. This was confirmed by decreases in ECAR and lactate formation, coupled with an increase in oxygen consumption rates in cancer cells. These findings collectively suggest that compound **12** represents a valuable metabolic modulator for the further development of anticancer drugs [66].

9. RS6212 (**13**, Figure 3). Pyrimidine derivatives showed significant in vitro cell growth inhibition of medulloblastoma or pancreatic tumor cells [67]. A tetrahydropyrimidine-5-carboxamide derivative that is able to induce cancer regression in mice bearing human tumors without significant side effects was recently patented [68]. RS6212 represents a novel specific pyridazine LDHA inhibitor, which was discovered, starting from the available enzyme crystal structure, through structure-based virtual screening studies based on docking and pharmacophore filters [69]. A preliminary in vitro test highlighted the ability of compound **13** to exhibit micromolar anticancer activity against Med1-MB (IC_50_ of 81 μM), a cell line that is dependent on aerobic glycolysis, as well as against HCT116 and SW620 CRC cell lines, A549 lung cancer, and PANC-1 pancreatic cancer cells. As an LDHA inhibitor, **13** was remarkably superior to compound **1**. Consistently, **13** increased the NADH content, decreased the lactate levels in tumor cells, and failed to inhibit the cell growth of HCT116 cells with LDHA deficiency. Compound **13** exhibited antitumor synergistic activity with rotenone, a proven mitochondrial complex I inhibitor that showed anti-carcinogenic activity in several studies [70].

### 2.2. Lipid Metabolism in Cancer

Lipids, a heterogenic class of hydrophobic molecules, constitute a diverse group of biomolecules encompassing fatty acids (FAs), glycerides (neutral glycerides and phosphoglycerides), and nonglyceride lipids (such as steroids and sphingolipids) [71]. The structural diversity inherent in their composition enables them to serve various functions; in fact, lipids represent key components of all cellular structures, serve as important signaling molecules, and play a pivotal role as energy sources to drive energy-demanding processes. Hence, the regulation of their metabolism (i.e., lipid uptake, modification, de novo synthesis, transport, and degradation) is evidently crucial for maintaining cellular homeostasis [72,73].

In 1953, Medes et al. [74] firstly observed that tumors convert glucose or acetate into lipids at a rate comparable to that seen in the liver; however, they also reported that the abovementioned mechanism is likely insufficiently rapid to meet the lipid demands of a swiftly growing tumor. Starting from these findings, following studies confirmed the importance of FA biosynthesis for cancer cell growth and survival [75].

Notably, lipid metabolic rewiring is a well-established hallmark of CRC cells, as well as many malignancies, undergoing transformation to a malignant phenotype [76]. Indeed, in response to increased demands for glucose, glutamine, and some amino acids, cancer cells, particularly CRC cells [77], adeptly use lipid metabolic reprogramming, which is characterized by high lipid levels and abnormal mitochondrial β-oxidation of FAs, to ensure the acquisition of both the energy stores and the fundamental components for biological membranes, which are necessary to support uncontrolled proliferation and survive in unfavorable environments that lack oxygen and nutrients [78,79]. Hence, the aberrant activation of lipid metabolism in cancer cells results in alterations in the membrane structure, the disruption of energy homeostasis, the modulation of cell signaling, changes in gene expression, and the redistribution of proteins. The alteration in the lipid metabolism, resulting in an increased amount of both lipid saturation and polyunsaturated FAs in membrane lipids, not only impacts cancer progression but also exerts an influence on crucial processes like endoplasmic reticulum (ER) stress and ferroptosis [80]; the latter was recently exploited as a therapeutic strategy for cancer treatment [81].

This strategic adaptation underpins critical processes, including proliferation, survival, invasion, metastasis, and responsiveness to the tumor microenvironment and cancer treatment. In a recent study, Zaytseva et al. [82] emphasized the importance of lipid dysregulation and its implication as a potential target for future therapies. They suggested that targeting FA metabolism could be a potent vulnerability worth exploring. Therefore, by inhibiting lipid biosynthesis, it becomes possible to restrict cancer cell survival and impede the growth of tumors [83].

**Figure 4:**
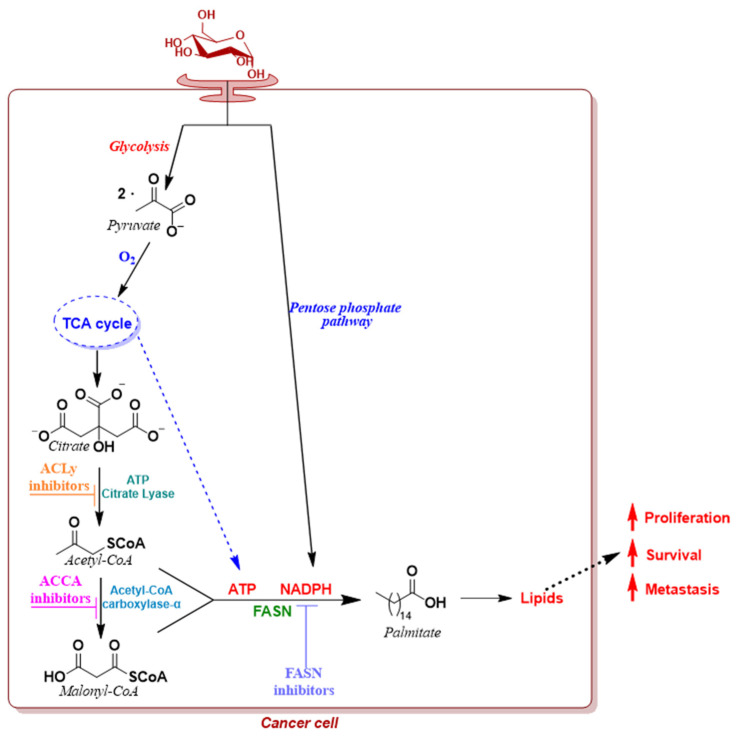
De novo fatty acid synthesis pathway functions in cancer cells.

This section intends to highlight the latest small molecules developed as potential therapeutic options for addressing altered lipid metabolism in CRC. These molecules aim to prevent the onset of cancer by specifically targeting pathways involved in the synthesis and storage of FAs (Figure 4) [84].

#### 2.2.1. Fatty Acid Synthase Inhibitors

Highly proliferative CRC cells exhibit an elevated demand for new building blocks, with a significant portion dedicated to the construction of cellular membranes. FAs are commonly used as building blocks for more complex lipids, including phospholipids, which, along with cholesterol, are the major constituents of cell membranes [78]. The upregulation of lipid metabolism is a prevalent feature in several solid malignancies. Often, increased de novo lipogenesis coincides with a heightened import of lipids from the extracellular space.

**Figure 5:**
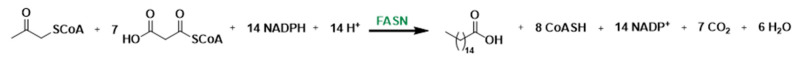
FASN-catalyzed reaction.

Fatty acid synthase (FASN) is a key enzymatic system that is capable of de novo long chain FA synthesis in mammals, and it is composed of two identical 272 kDa multifunctional polypeptides with seven functional domains each. Its main function is to catalyze the conversion, in the presence of NADPH, of acetyl-coenzyme A (CoA), a by-product of the Krebs cycle reaction to yield ATP, and malonyl-CoA to obtain fatty acid palmitate (Figure 5). In addition, FASN regulates the tumor vasculature by inducing secretion angiogenic factors [85].

In various tumor types, such as colon, stomach, breast, lung, prostate, and ovarian carcinomas, elevated levels of FASN expression have been observed in comparison to normal tissues. Given the observed upregulation of FASN and the increased saturation of membrane lipids in CRC, both of which are linked to tumor growth and metastasis [76], and considering the essential role of de novo lipogenesis in CRC tumor growth and survival, the most straightforward approach to lower the fatty acid levels in cancer cells is through the inhibition of FASN. Therefore, cancer cell proliferation can be restricted by inhibiting enzymes and reducing the availability of fatty acids in the diet.

In this review, several FASN inhibitors developed to date have been reported. Among them, some have demonstrated good preclinical antitumor activity in cancer cell lines and xenograft models, while a few have successfully progressed from preclinical studies to clinical trials, emerging as promising candidates for CRC treatment.

**Figure 6:**
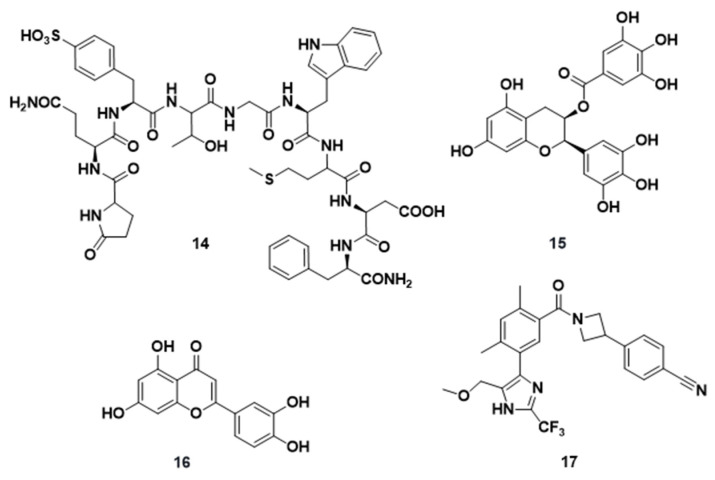
Chemical structures of compounds **14**–**17** as fatty acid synthase inhibitors.

1. Cerulein ((2S,3R)-2,3-epoxy-4-oxo-7,10-dodecadienamide, **14**, Figure 6) is a natural decapeptide that is isolated from Cephalosporium caerulens, and it is endowed with structural elements common to the natural gastrointestinal peptide hormone cholecystokinin, which stimulates secretions on the stomach, bile, pancreas, and on some smooth muscles. It was the first antibiotic reported to inhibit FASN [86,87]. Compound **14** induced apoptosis in both Colon 26 and CMT 93 murine CRC cell lines. In Colon-26 cells, **14** significantly reduced the numbers and sizes of liver metastatic CRC tumors, and its activity was associated with reduced levels of phosphorylated Akt [88].

It is well known that oxaliplatin, a commonly used drug in platinum-based chemotherapy for unresectable CRC, can induce peripheral neurotoxicity as a comprehensive adverse reaction [89]. In a study conducted by Murata et al., it was revealed that compound **14**, in combination with oxaliplatin, demonstrated a synergistic effect in vitro and in vivo, which led to the reduction in the oxaliplatin dosage, making it possible for the patient to endure chemotherapy over a longer period [90]. Indeed, the combination of **14** with oxaliplatin induced the p53-p21 pathway and p38 activation, which causes cell cycle arrest, and induced caspase-3 cleavage at a smaller concentration than **14** or oxaliplatin alone, finally causing apoptosis. In xenotransplanted immune-deficient (SCID) mice, the group treated with the **14** and oxaliplatin drug combination significantly inhibited tumor progression compared to the control groups that were treated with **14** or oxaliplatin [91]. However, it was reported that **14** activates both NADPH oxidase, which is a major source of reactive oxygen species during inflammation and apoptosis in pancreatic acinar cells, and the Janus kinase pathway, underlying the pathogenesis of acute pancreatitis [92].

2. (-)-Epigallocatechin gallate (EGCG, **15**, Figure 6), formally the ester of gallic acid with the (3R)-hydroxy group of (-)-epigallocatechin, is a polyphenol extracted from green tea and has been proven to be a potent inhibitor of FASN, along with (-)-gallocatechin gallate, another component of green tea extract, whereas ungallated catechins showed a weaker inhibition [93]. In a study that aimed to investigate the effects in CRC SW480, SW620, and LS411N cell lines, **15** significantly inhibited cell proliferation and induced apoptosis through the activation of apoptosis-related proteins, such as caspase-3, caspase-9, and PARP-1. Signaling and transcriptional activation factor 3 (STAT3) is involved in cancer propagation: it activates the transcription of downstream target genes, inhibits apoptosis, and regulates members of the Bcl-2 family [94,95]. In the three cell lines, **15** significantly downregulated both STAT3 and phosphorylated STAT3 (p-STAT3); decreased Bcl-2, MCL-1, and Vimentin; and increased E-cadherin [96]. Additionally, **15** showed to downregulate STAT3 expression in breast cancer stem cell phenotype MDA-MB-231 cells and MCF7 cells [97] and in nasopharyngeal carcinoma [98].

In vitro and in vivo studies carried out by Jin et al. [99] confirmed the ability of compound **15** to inhibit the proliferation of CRC cells by inducing apoptosis in a dose-dependent manner and influencing the cell cycle. Furthermore, while the exact mechanism necessitates further studies, the authors demonstrated that compound **15** inhibited CRC cell proliferation by suppressing the expressions of HES1 and Notch2, which are genes associated with the Notch signaling pathway implicated in malignant transformation.

While in vivo studies showed the potential of compound **15** to induce apoptosis and cell cycle arrest at the G_2_/M phase, its instability and rapid metabolism, attributed to the presence of hydroxyl groups on phenol rings, suggest an ineffective local concentration within tumors, thereby limiting its clinical application. Therefore, to increase its pharmacokinetic properties by oral route, several nanoformulations of compound **15** emerged as alternatives to the traditional one. In their study, Granja et al. [100] employed a nanomedicine-based strategy by developing folic acid-functionalized nanostructured lipid carriers that load compound **15**, providing enhanced physicochemical characteristics for oral administration.

In addition, combining compound **15** with other drugs may be a more effective strategy for restoring drug sensitivity and achieving a balance in cytotoxicity. Of note, it has been demonstrated that compound **15** sensitizes chemoresistant CRC cells to 5-fluorouracil (5-FU) by targeting the cancer stem cell population. Indeed, Toden et al. [101] reported that the treatment of 5-FU resistant cells with compound **15** resulted in the suppression of spheroid-derived cancer stem cell formation. This suggests that **15** has the potential to prevent the development of cancer stem cells in CRC, highlighting its potential as a supportive treatment for patients with CRC.

Encouraging preclinical outcomes have prompted the initiation of clinical trials examining compound **15** supplementation as a potential therapeutic option for patients with CRC. While the findings endorse the notion that compound **15** might exhibit certain chemopreventive effects for patients with CRC, further investigations involving larger cohorts and extended study durations are required to substantiate this effect [102].

3. Luteolin (3′,4′,5,7-tetrahydroxyflavone, **16**, Figure 6) is a naturally occurring 4H-chromen-4-one derivative belonging to the family of flavonoids that exhibits several therapeutic properties, such as anti-proliferative, antioxidant, anti-inflammatory, and anti-infective activities. Compound **16** showed anticancer activity against CRC by interfering with the Wnt/β-catenin signaling pathway [103] and by reducing oxidative stress [104]. Compound **16** proved to be a potent FASN inhibitor capable of reducing DNA synthesis and inhibiting HT-29 cell growth and proliferation through the induction of cell cycle arrest at the G_1_ and G_2_/M phases, leading to a dose-dependent apoptotic effect. In addition, compound **16** upregulated microRNA-384, a short non-coding RNA that is involved in the post-transcriptional regulation of gene expression, and it downregulated the expression of pleiotrophin, a potent mitogenic cytokine [105], both in CRC cells and tissues, suggesting that the anticancer activity exerted by **16** against CRC cells went through the modulation of the heparin-binding growth factor pleiotrophin (PTN) via microRNA 384 [106]. Apoptosis induced by **16** was mediated through the expression of death receptor 5 (DR5), the cleavage of Bcl-2-interacting domain, and the activation of caspases 3 and 9–10, suggesting that DR5 plays a role in **16**-induced apoptosis [107].
4. TVB-3664 (**17**, Figure 6) is a potent FASN inhibitor developed by 3-V Biosciences. TVB-3664 demonstrated anti-tumor activity in multiple cancers, including CRC, in vitro and in vivo and has a favorable tolerability profile in phase I clinical trials [108,109]. CRC cells and patient-derived xenografts (PDXs) from patients with CRC have shown a wide range of sensitivity to FASN inhibitors. In nine CRC cells (PDXs), treatment with **17** yielded a significant reduction in the tumor volume in 30% of cases. The anti-tumor treatment with **17** caused significant reductions in the pool of adenine nucleotides, which play key metabolic functions [110], and in lipids, fatty acids, and phospholipids and caused an increase in sphingolipids.

Of note, in a subsequent study published by the same authors [111], the link between FASN and Fatty Acid Translocase (CD36) in the setting of CRC was described for the first time. CD36, a multifunctional transmembrane glycoprotein, plays a key role in FA metabolism. CD36 translocation to the plasma membrane allows its extracellular domain to bind low-density lipoproteins, facilitating their transport across the plasma membrane into the cytosol [112]. This process is involved in the metabolism of extracellular fatty acids; therefore, CD36 may undergo various types of post-translational modifications. Zaytseva et al. reported that CD36 protein is highly expressed in CRC and primarily localized to the plasma membrane when FASN is inhibited by compound **17** in Pt 93 and Pt 130 cells [111]. Furthermore, to investigate the potential improvement in the efficacy of compound **17** through CD36 inhibition, primary CRC cells from Pt 93 and Pt 130 were subjected to treatment with the CD36 inhibitor sulfosuccinimidyl oleate and the FASN inhibitor compound **17**. These treatments were administered individually and in combination under normal and serum-starved media conditions. The results reveal that there was a significant reduction in cellular proliferation when each compound was administered individually. Moreover, the combination of both compounds further amplified this reduction, indicating that concurrent treatment with CD36 inhibition is crucial for optimizing the effectiveness of FASN-targeted therapy. Additionally, the major oncogenic pathways affected by compound **17** include Akt, Erk1/2, and AMPK. Notably, the elevation in the pAkt and survivin levels has been linked to the tumor-promoting effect associated with the upregulation of CD36 [111,113].

#### 2.2.2. ATP-Citrate Lyase Inhibitors

**Figure 7:**
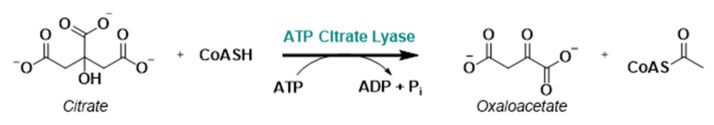
ACLy-catalyzed reaction.

ATP citrate lyase (ACLy) is an important, ubiquitous homotetrameric cytosolic enzyme involved in de novo lipogenesis, and it is responsible for the production of non-mitochondrial acetyl-CoA [114]. More precisely, it serves as a link between the tricarboxylic cycle and lipogenesis by catalyzing the conversion of citrate (originating in the mitochondria) and CoA to acetyl-CoA (a precursor for both FAs and cholesterol synthesis) and oxaloacetate in the presence of ATP (Figure 7).

Enhanced de novo lipogenesis is one of the major metabolic alterations used by cancer cells to sustain survival and growth [115]. ACLy has been found to be upregulated in resistant CRC cells and, in general, in the glycolytic phenotype of tumors; thus, ACLy has become a novel target for cancer therapeutics [116]. The simultaneous activations of the pathways of ACLy and PI3K/Akt, the latter of which regulates both glucose and lipid metabolism as well as ACLy levels and activity after being phosphorylated [117], were identified as being crucial in conferring resistance to the topoisomerase I inhibitor SN38 in CRC cells. Notably, inhibiting ACLy was found to enhance the sensitivity of chemo-naïve HT29 cells to SN38 treatment [118].

The increasing evidence underscoring the significance of ACLy in tumor cell growth and metabolic disorders has prompted this review to consolidate and summarize the most promising ACLy inhibitors developed to date.

**Figure 8:**
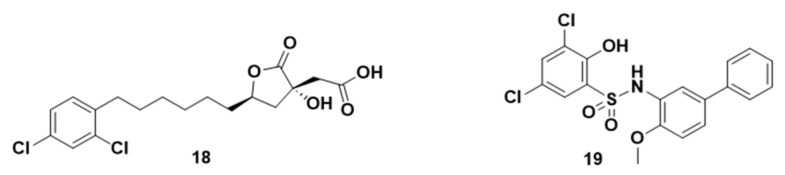
Chemical structures of compounds **18** and **19** as ATP-citrate lyase inhibitors.

1. SB204990 (**18**, Figure 8), a prodrug derived after the lactonization of the hydroxy-acid SB201076, represents a potent and specific ACLy inhibitor. In 2005, Hatzivassiliou et al. [119] demonstrated the ability of ACLy inhibition to suppress tumor growth, correlating with the glycolytic phenotype of the tumor. Their study included three human tumor cell lines—A549, PC3, and SKOV3—treated with compound **18**, revealing dose- and time-dependent sensitivity. This treatment resulted in a notable reduction in the total acetyl-CoA level across all three treated cells compared to the controls. Notably, the SKOV3 cells exhibited resistance to compound **18**, which was possibly attributed to their lower glycolysis rate compared to the other cell lines, resulting in reduced glucose utilization and lactate production.

Furthermore, the authors assessed the in vivo antitumor activity of compound **18** using A549, PC3, and SKOV3 xenograft models in nude mice. In comparison to treatment with the vehicle alone, this treatment demonstrated significant antineoplastic cytostatic activity against both A549 and PC3 xenografts. However, no effect was observed in the SKOV3 xenografts.

Finally, the in vivo bioactivation of compound **18** into active SB201076 was confirmed through oral administration to rats. After a 7-day treatment, it demonstrated significant reductions in plasma cholesterol (up to 46%) and triglycerides (up to 80%). Similar effects were observed in dogs, although over a longer treatment period of 15 days, albeit with less pronounced reductions [120]. In the case of dogs, the cholesterol levels were reduced by up to 23%, and the triglyceride levels were reduced by up to 38%.

2. GSK165 (2-hydroxy-N-arylbenzenesulfonamide, **19**, Figure 8) was identified as a novel ACLy inhibitor with an in vitro IC_50_ value of 0.13 μM. In high-fat-diet-fed mice, **19** lowered plasma cholesterol, triglyceride, and glucose and inhibited weight gain [121]. Zhou et al. [118] reported that compound **19** exhibited growth inhibition in cancer cells as a standalone agent, with an IC_50_ value of ~30 μM, whereas at a concentration of 40 μM, compound **19** demonstrated its ability to sensitize HT29 cells to SN38.

#### 2.2.3. Acetyl-CoA Carboxylase-α Inhibitors

**Figure 9:**
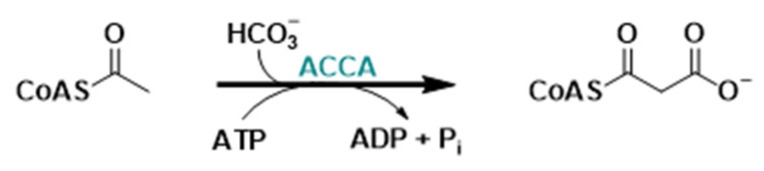
ACCA-catalyzed reaction.

Acetyl-CoA carboxylase-α (ACCA), a biotin-dependent multi-domain enzyme, represents the starting point of de novo lipogenesis; in fact, in the presence of bicarbonate as a CO_2_ donor, it catalyzes the irreversible carboxylation of cytosolic acetyl-CoA into malonyl-CoA (Figure 9), which is a crucial substrate for FA synthesis catalyzation by FASN.

Interestingly, the upregulation of the lipogenic enzyme isoform ACCA has been implicated in the initiation and development of numerous malignancies, and it has been documented in several types of human cancers, including colon, breast, prostate, ovary, lung, and endometrial cancers [122,123,124]. Therefore, ACCA inhibition has become an appealing challenge in the pursuit of developing new strategies for anti-tumor treatments.

**Figure 10:**
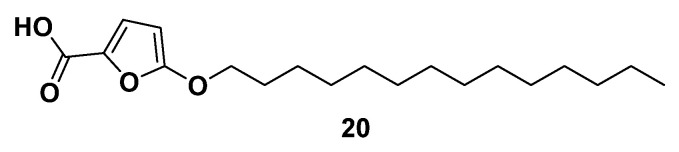
Chemical structure of compound **20** as acetyl-CoA carboxylase-α inhibitor.

1. TOFA (5-tetradecyloxy-2-furoic acid, **20**, Figure 10) is an allosteric inhibitor of the ACCA that represents the rate-limiting enzyme of the FA synthesis pathway. More precisely, inside adipocytes and hepatocytes, TOFA is converted to TOFyl-CoA (5-tetradecyloxy-2-furoyl-CoA), exerting the allosteric inhibition on ACCA [125]. It showed strong cytotoxicity and induced apoptosis in human NCI-H460 lung cancer, HCT-8, and HCT-15 colorectal cancer cells exposed to **20** in a dose-dependent fashion [126,127]. Moreover, it was reported that compound **20** inhibited fatty acid synthesis, increased the oxidation of fatty acid and ketogenesis, and decreased the synthesis of triglycerides and the production of very low-density lipoprotein (VLD) [128]. In addition, compound **20** inhibited the growth of ovarian cancer cell lines COC1 and COC1/DDP; arrested the cells in the G_0_/G_1_ cell cycle phase; induced apoptosis; inhibited the expressions of Cyclin D1, CDK4, and Bcl-2 proteins; and activated cleaved caspase-3 [129].

Taken together, these results imply that compound **20** could serve as a good starting point for the development of novel and highly sensitive ACCA inhibitors as promising antitumor agents.

### 2.3. Glutaminolysis in Cancer

Glutamine, the most abundant amino acid in the body, represents an important source of nitrogen for nonessential amino acid and nucleotide (purines and pyrimidines) biosynthesis due to the presence of two nitrogen side chains (an amino and an amide group) in its structure. Beyond glucose, glutamine is the other major substrate playing a pivotal role in cellular energy metabolism. In addition, it takes part in macromolecular biosynthesis, regulates cellular redox homeostasis, maintains mitochondrial metabolism, and supports the proliferation of tumor cells [130].

In the previous section of the present review, the importance of metabolic reprogramming in tumorigenesis and cancer progression was described. While increased aerobic glycolysis or lipogenesis alone cannot fully satisfy the metabolic demands of proliferating cancer cells, it has been observed that, to fuel their bioenergetic and biosynthetic demands and maintain a functional tricarboxylic acid (TCA) cycle, cancer cells exhibit elevated glutamine metabolism, known as glutaminolysis, along with a heightened glucose metabolism [131].

**Figure 11:**
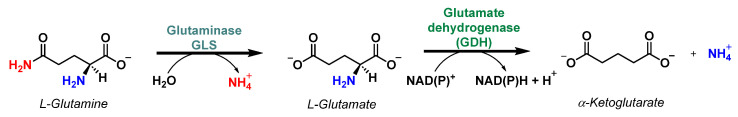
GLS and GDH catalyzed reactions.

Glutaminolysis initiates with the uptake of exogenous glutamine into the cytoplasm via the upregulation of glutamine transporter expression (SLC1A5 and SLC7A5) in several cancer cells [132]. Within the cell, the mitochondrial enzyme glutaminase 1 (GLS1 or GLS) catalyzes the hydrolysis of glutamine to glutamate and ammonium. Glutamate is subsequently deaminated, by glutamate dehydrogenase (GLUD1), to ammonium and α-ketoglutarate (α-KG), the latter of which is channeled into the TCA cycle (Figure 11).

In rapidly dividing cells, this sequence of reactions holds particular significance. A substantial amount of the TCA cycle metabolite citrate is exported from mitochondria to produce cytosolic acetyl-CoA, which is crucial for lipid biosynthesis [133]. To maintain TCA cycle intermediates (anaplerosis), replenishment becomes essential, and glutamine frequently plays a pivotal role as the primary anaplerotic substrate, converting to α-KG via glutamate.

Moreover, tumor cells harbor large intracellular pools of glutamate, since GLS is highly expressed in several tumors, maintaining the ability to convert glutamine into glutamate [130]. Evidence demonstrates that GLS activity correlates with tumor growth rates in vivo [134,135], and various genetic factors, such as Myc family members, are implicated in the regulation of both GLS expression and glutamine metabolism.

#### Glutaminolysis Inhibitors

**Figure 12:**
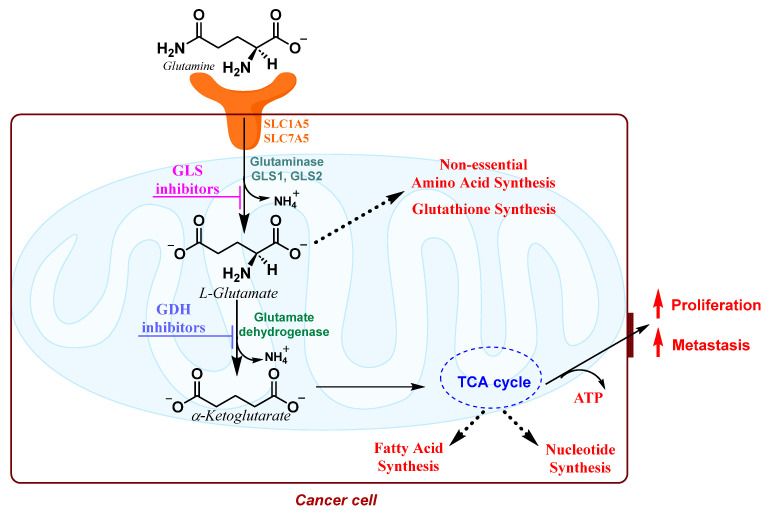
Glutamine metabolic pathways in cancer cells.

Glutamine plays a crucial role as a significant nitrogen and carbon source for actively proliferating cells, particularly in glutamine-addicted cancer cells. It serves as an essential building block for various biosynthetic processes, including the synthesis of glutathione, NADPH, nucleotides, and fatty acids (Figure 12).

Yuneva et al. highlighted in their work that MYC expression in cancer cells resulted in a strong dependence on glutamine. Indeed, they demonstrated that the removal of glutamine induced cell apoptosis primarily due to the stimulation of glutaminolysis by an MYC-regulated transcriptional program [136]. Consequently, MYC induces a reliance on glutamine for the mitochondrial TCA cycle [137]. MYC also activates key glutamine transporters, including SLC38A5 and SLC1A5 (ASCT2), the latter being involved in glutamine-dependent mTORC1 activation. Elevated levels of mTORC1, a regulator of protein translation responsive to cellular glutamine levels, are observed in glutamine-dependent cells. Furthermore, in cells that are dependent on glutamine, higher-than-average levels of SLC1A5 are often present [138]. Moreover, it was discovered that cells with overexpressed MYC exhibit higher levels of glutaminase compared to those with low MYC expression [36]. Thus, it is reasonable to assume that all of these factors (i.e., MYC, SLC1A5, mTORC1, and glutaminase) might potentially serve as biomarkers in future clinical trials for evaluating the efficacy of glutaminolysis inhibitors as they are more pronounced in glutamine-dependent cells.

Therefore, glutaminolysis has been suggested as a hallmark of cancer metabolism [139], drawing increased research attention for the development of novel small molecules that act as glutaminolysis inhibitors to treat cancer. In this regard, recent evidence reported that the inhibition of the glutaminase activity caused a reduction in tumor growth cells [140,141].

However, it must be mentioned the emergence of resistance to some drugs targeting glutaminolysis has been observed. Therefore, at the same time, there is an urgent need to develop alternative strategies to overcome this serious issue. One approach currently employed to address the risk of resistance involves the use of synergistic drug combinations, enhancing the effectiveness of tumor chemotherapy while reducing drug resistance and side effects.

Here, we report a novel class of metabolism-targeted small molecules that inhibit GLS isoforms that are not commonly expressed in normal cells.

**Figure 13:**
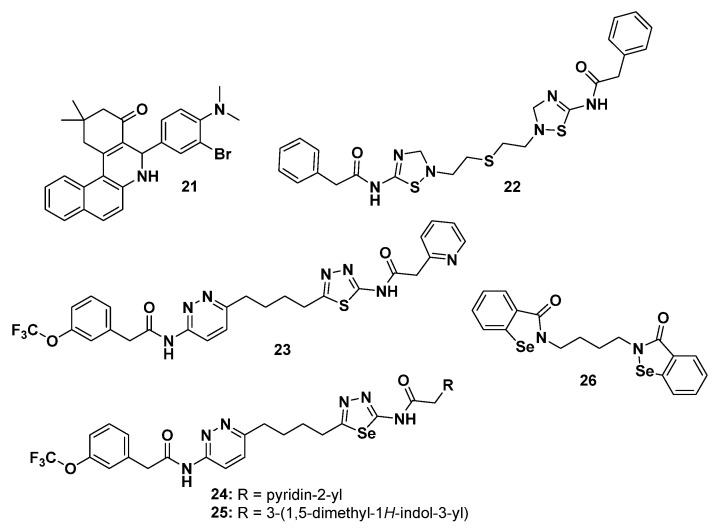
Chemical structures of compounds **21**–**26** as glutaminolysis inhibitors.

1. Compound 968 (**21**, Figure 13) is a small molecule that acts as an allosteric regulator of isoform 1 of glutaminase (GLS1) and inhibits the activity of both major splice GLS variants: long form KGA and short form GAC [142]. Compound **21** demonstrated antitumor activity in vitro in several types of cancer, including lymphoma, breast cancer, glioblastoma, and lung cancer [143,144,145]. In fibroblasts, **21** inhibited oncogenic transformation caused by various Rho family GTPases without exhibiting toxic effects on normal cells [146]. Compound **21** inhibited the proliferation of HEY, SKOV3, and IGROV-1 cells upon 5-day treatment and induced apoptosis [147]. In addition, **21**, in combination with a low concentration of paclitaxel, showed stronger inhibitory effects. The treatment of endometrial cancer with **21** downregulated the expressions of GLS1 and cyclin D1 and upregulated the expressions of P21 and E-cadherin. In xenograft mouse models of endometrial cancer, **21** significantly suppressed tumor growth [148]. The combination of compound **21** with metformin effectively suppressed CSCs in SW620 cells, and this effect was further enhanced in HT29 cells. Notably, SW620 cells exhibited higher expression levels of GLU1 and the glutamine transporter ASCT2 compared to HT29 cells [149].
2. BPTES (bis-2-(5-phenylacetamido-1,2,4-thiadiazol-2-yl)ethyl sulfide, **22**, Figure 13) was reported as a potent and allosterically selective inhibitor of GLS, consequently inhibiting glutaminolysis. A crystal structure analysis showed that compound **22** binds to the dimer interface of GLS, consequently stabilizing the inactive tetrameric form of the enzyme [150]. Compound **22** at 300 nM decreased the glutamate in intestinal epithelial and neuronal cells with highly specific binding to the enzyme and enzyme–substrate complex to form a stable but inactive tetramer. Despite its poor physiochemical properties, such as its poor aqueous solubility, it has been used as key molecular template for the identification of other allosteric GLS inhibitors with more potent and enhanced drug-like properties.

Moreover, Gowda et al. [151] reported that compound **22** induced metabolism changes in MCF7 and TNBC MDA-MB231 cell lines and formed 41 unique metabolites, which were especially pronounced under hypoxic conditions, as a result of the alteration in several metabolic pathways including glutamine metabolism, glycolysis, TCA cycle, and amino acid pathways.

In a recent study carried out by Liu et al. [152], an upregulation of GLS expression was observed in CRC. Consequently, the depletion of GLS expression or the inhibition of its activity led to the blockage of CRC cell proliferation and migration. Notably, they observed that the depletion of GLS induced by compound **22** in CRC cells was associated with an increase in reactive oxygen species (ROS) production and the activation of the redox/Nrf2/autophagy pathway. These findings reveal an autophagic regulatory mechanism in CRC metastasis, emphasizing the potential of targeting GLS1 activity as a therapeutic strategy for treating colorectal cancer.

3. CB-839 (**23**, Figure 13) is a potent and selective inhibitor of glutaminase that showed in vitro antitumor activity against a panel of TNBC cell lines. Compound **23** showed in vivo efficacy in breast cancer xenograft models, both alone and in combination with paclitaxel [153]. Compound **23** exhibited cytotoxicity in tumor-infiltrating lymphocytes (TILs) isolated from patient-derived melanoma cells. Notably, it demonstrated a more potent decrease in the conversion of glutamine to α-KG in tumor cells compared to TILs in co-cultures. This suggests that **23** may have the potential to selectively modulate the metabolism of tumor and immune cells, potentially enhancing immune function in the tumor microenvironment. In vivo treatment with compound **23** activated melanoma antigen-specific T cells. Furthermore, in combination with anti-PD1 or anti-CTLA4 antibodies, it synergized their activity [154].

Compound **23** exhibited inhibitory effects on the growth of a xenograft tumor model carrying the PIK3CA mutation, which renders CRC more dependent on glutamine [155] compared to wild-type CRC. Finally, the combination of **23** with 5-FU induced regression of PIK3CA-mutant tumors in xenograft models, when tested with the carbamate ester prodrug of 5-FU, capecitabine, was active at biological doses and demonstrated good tolerance in a phase I clinical trial [156].

4. GPD-20 (**24**, Figure 13) and GPD-23 (**25**, Figure 13) are selenadiazole analogues of compound **23**. They showed an enhanced inhibition of cancer cells and of the KGA isoform, demonstrated higher potency in inducing ROS, and showed better cell accumulation. However, in both the glutamine-dependent HCT116 and aggressive H22 liver cancer xenograft models, **24**–**25** only resulted in a partial reduction in the tumor size [157,158].
5. CDP-3B (Hexylselen, **26**, Figure 13) is a seleno derivative inhibitor of KGA/glutamate dehydrogenase (GDH) that completely inhibited the cancer cell growth of many aggressive tumors, reduced the tumor size in an aggressive liver cancer xenograft model, and showed no toxic effects on normal cells at a concentration of up to 10 μM. Compound **26** also inhibited thioredoxin reductase (TrxR) and glutamine amidotransferase (GatCAB), thus affecting the Akt/Erk/caspase-9 signaling pathways [159].

### 2.4. Other Target Inhibitors

**Figure 14:**
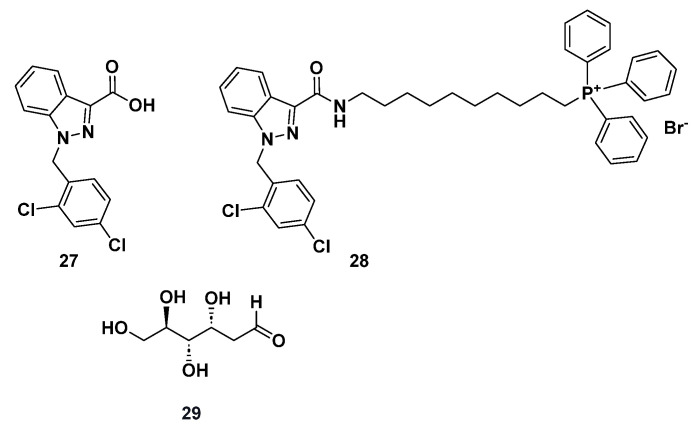
Chemical structures of inhibitors **27**–**29**.

1. Lonidamine (**27**, Figure 14) is a derivative of indazole-3-carboxylic acid that blocks glycolysis in cancer cells, resulting in a reduction in cellular ATP. It has progressed into clinical trials and gained approval for the treatment of lung, breast, prostate, and brain cancers either alone or in combination with other anticancer agents. Compound **27** has been explored for the treatment of several types of cancer, including CRC [160].

Compound **27** inhibits the energy metabolism in tumor cells [161], particularly aerobic glycolysis, by interfering with the mitochondrially bound hexokinase that phosphorylates glucose to glucose 6-phosphate [162,163]. It also induces ROS formation by inhibiting the succinate-ubiquinone reductase activity of respiratory complex II [164].

Of note, compound **27** is commonly employed to enhance the sensitivity of chemotherapeutic drugs, since it disrupts energy metabolism associated with chemo- or radio-resistance [165]. Furthermore, a strategic modification involving the attachment of a covalently linked lipophilic cation, such as the triphenyl phosphonium cation (TPP^+^), has been pursued. This chemical alteration has increased the lipophilicity and cellular uptake of various bioactive agents, allowing for nearly a 1000-fold higher mitochondrial drug concentration [166].

2. Mito-lonidamine (**28**, Figure 14) was synthesized by conjugating compound **27** with TPP^+^ via an aliphatic chain linker to enhance its potential mitochondrial activity. At low micromolar concentrations, compound **28** was more effective than **27** in primary lung tumors and lung cancer brain metastases in an orthotopic mouse model. It inhibited mitochondrial complexes I and II and stimulated ROS production through the induction of autophagic cell death [163]. In a phase II study involving patients with metastatic colorectal adenocarcinoma who had undergone previous chemotherapy for metastatic disease, the majority of those treated with compound **28** exhibited incomplete or partial remissions [167].
3. 2-deoxy-D-glucose (2-DG, **29**, Figure 14) has demonstrated the ability to enhance the antitumor effects of radio- and chemotherapy both in vitro and in vivo across various solid tumors, including lung, breast, pancreas, head, neck, and gastric tumors, with weak damage to normal cells [168]. In cancer cells under hypoxic conditions, **29** is recognized as a glycolysis inhibitor that hampers ATP production. When applied to chemoresistant CRC cells expressing drug resistance-related proteins, compound **29** treatment led to a reduced expression of glycolysis-related enzymes and the secretion of cytokines associated with epithelial-to-mesenchymal transition (EMT) [169]. Moreover, **29** inhibited the activation of A disintegrin and metalloproteinase (ADAM)10 and, potentially, ADAM17, both of which play a central role in generating soluble CD137 (sCD137), which is considered a splice variant of CD137 within the TNF receptor family [170].

Notably, accumulating evidence shows that **29** was more toxic than the glycolytic inhibitor 2-fluorodeoxy-D-glucose in select tumor cell lines growing under normoxia. It was found that in **29**-sensitive cancer cells, under normoxic conditions, a stronger inhibition of the N-linked glycosylation occurred (but not glycolysis). These findings open the way to use **29** as single anticancer agent in solid tumors in both aerobic (via N-linked glycosylation) and hypoxic (via glycolysis) conditions [171].

## 3. Conclusions

Alterations in cellular metabolism, including dysregulation in glycolysis, lipid metabolism, and glutaminolysis, which are widely recognized as hallmarks of cancer, play pivotal roles in tumor growth and metastasis [172]. Consequently, the incessant pursuit of novel and effective anticancer treatments has led to the development of small molecule inhibitors, summarized in Table 1, precisely targeting these aberrant metabolic pathways. However, given the heterogeneity of CRC, the need to identify robust biomarkers associated with specific metabolic alterations is becoming imperative in order to develop personalized treatment strategies. Therefore, we strongly believe that ongoing research in this field has the potential to expand the arsenal of treatments that are currently available and to overcome multi-drug resistance mechanisms by also exploring the synergistic effects achievable through the strategic combination of different inhibitors targeting multiple metabolic pathways.

## Figures and Tables

**Table 1:** Compounds targeting lactate dehydrogenase, lipid metabolism, and glutaminolysis in CRC.

Category	Compound	Targets	Ref.
	Oxamate	LDHA, AAT	[36,37,38,39,40]
	Gossypol	LDH, GA3PDH, MDH, ICDH	[42]
	FX11 with Daporinad	LDHA, NMPRTase	[49,51]
	Galloflavin	LDHA, LDHB	[53]
	Quinoline-3-sulfonamide	LDHA, PKM2 ^1^	[50]
	*N*-Hydroxyindole-2-carboxylate derivatives	hLDH5	[59,60]
	NCATS-SM1441	LDHA, LDHB	[64]
	CHK-336	LDHA	[65]
	Pyran-4-one	LDHA	[66]
	Pyrimidine-5-carboxamide	LDHA	[68] ^2^
	RS6212	LDHA	[70]
Lipid metabolism	Cerulein	FASN, Akt ^1^	[86,87,88]
	Cerulein, oxaliplatin	Induction p53-p21, p38	[91]
	(-)-Epigallocatechin gallate	FASN, STAT3	[93,96]
	Luteolin	Wnt/β-catenin, PTN	[104,106]
	TVB-3664	FASN, Akt ^1^, Erk1/2 ^1^, AMPK ^1^	[108,109,113]
	SB204990	ACLy	[119]
	GSK165	ACLy	[121]
	TOFA	ACCA	[126,127]
Glutaminolysis	Compd 968	GLS1 KGA GAC, Rho ^1^	[142,146]
	Compd 968, metformin	GLS1, ASCT2 ^1^	[149]
	BTES	GLS1 KGA	[151,173]
	CB-839	GLS, αKGA	[153]
	CB-839, capecitabine	GLS, 5-FU target	[156]
	CDP-3B	KGA, GDH, TrxR, GatCAB	[159]
Other	Lonidamine	Hexokinase, complex II	[162,164]
	Mito-lonidamine	Hexokinase, complex I and II	[163,167]
	2-deoxy-*D*-glucose	Glycolysis, sCD1371	[169]

^1^ Indirect activity; ^2^ patent.

## Data Availability

Not applicable.

## Abbreviations

5-FU	5-fluorouracil
α-KG	α-ketoglutarate
ACACA	acetyl-CoA carboxylase alpha
ACLy	ATP-citrate lyase
ADAM	A disintegrin and metalloproteinase
ATP	adenosine triphosphate
BRAF	B-Raf protooncogene
CCR	colon-rectal cancer
CoA	coenzyme A
CRC	colorectal cancer
CSCs	cancer stem cells
DMEs	drug-metabolizing enzymes
ECAR	extracellular acidification rate
ER	endoplasmic reticulum
FAs	fatty acids
FASN	fatty acid synthase
GA3PDH	glucelaldehyde-3-phosphate dehydrogenase
GatCAB	glutamine amidotransferase
GDH	glutamate dehydrogenase
GLS	glutaminase
GLS1	glutaminase isoform 1
GLUD1	glutamate dehydrogenase
GMB	glioblastoma
GnRH	gonadotropin-releasing hormone
GST	glycolysis stress test
HCC	hepatocellular carcinoma
HIF-1	hypoxia-inducible factor 1
HIT	heparin-induced thrombocytopenia
ICDH	isocitrate dehydrogenase
KGA	kidney mitochondrial glutaminase
KRAS	Kirsten rat sarcoma viral oncogene homolog
LDH	lactate dehydrogenase
LDHA	lactate dehydrogenase A
LDHB	lactate dehydrogenase B
mCRC	metastatic colorectal cancer
MDH	malate dehydrogenase
miRNA-106a	microRNA-106a
NSCLC	non-small-cell lung cancer
OxPhos	oxidative phosphorylation
PH1	primary hyperoxaluria type 1
PK	pharmacokinetic
PPAR	peroxisome-proliferator activated receptor
p-STAT3	phosphorylated STAT3
ROS	reactive oxygen species
STAT3	signaling and transcriptional activation factor 3
TCA	tricarboxylic acid
TPP+	triphenyl phosphonium cation
TrxR	thioredoxin reductase
VLDL	very-low-density lipoprotein
